# Psychological Burden in Meningioma Patients under a Wait-and-Watch Strategy and after Complete Resection Is High—Results of a Prospective Single Center Study

**DOI:** 10.3390/cancers12123503

**Published:** 2020-11-25

**Authors:** Darius Kalasauskas, Naureen Keric, Salman Abu Ajaj, Leoni von Cube, Florian Ringel, Mirjam Renovanz

**Affiliations:** 1 Department of Neurosurgery, University Medical Centre, Johannes Gutenberg University Mainz, Langenbeckstr. 1, 55131 Mainz, Germany; Naureen.keric@unimedizin-mainz.de (N.K.); sabuajaj@students.uni-mainz.de (S.A.A.); lvoncube@students.uni-mainz.de (L.v.C.); Florian.Ringel@unimedizin-mainz.de (F.R.); mirjam.renovanz@med.uni-tuebingen.de (M.R.); 2 Department of Neurosurgery, University Hospital Tübingen, Eberhard Karls University Tübingen, Hoppe-Seyler-Straße 3, 72076 Tübingen, Germany; 3 Department of Neurology & Interdisciplinary Neuro-Oncology, University Hospital Tübingen, Hertie Institute for Clinical Brain Research, Otfried-Müller-Straße 27, 72076 Tübingen, Germany

**Keywords:** meningioma, quality of life, psychological distress, anxiety, depression, surgery, wait-and-watch

## Abstract

**Simple Summary:**

Asymptomatic meningiomas are found in 1–2% of cranial MRIs. Most of them demonstrate no or minimal growth and are observed with follow-up imaging. However, the patients face a diagnosis of a brain tumor. So far, there is no established distress screening for such patients. In this study, we evaluated the psychological burden of patients with small asymptomatic meningiomas and compared it with patients after complete meningioma resection and excellent postoperative outcome. We found a high prevalence of anxiety and depression symptoms in both study groups. This demonstrates that even patients with benign asymptomatic intracranial tumors might be under significant distress and need psychooncological support.

**Abstract:**

The diagnosis of intracranial meningiomas as incidental findings is increasing by growing availability of MRI diagnostics. However, the psychological distress of patients with incidental meningiomas under a wait-and-watch strategy is unknown. Therefore, we aimed to compare the psychosocial situation of meningioma patients under wait-and-watch to patients after complete resection to bridge this gap. The inclusion criteria for the prospective monocenter study were either an incidental meningioma under a wait-and-watch strategy or no neurologic deficits after complete resection. Sociodemographic, clinical, and health-related quality of life and clinical data were assessed. Psychosocial factors were measured by the Distress Thermometer (DT), Hospital Anxiety and Depression Scale (HADS), Brief Fatigue Inventory (BFI), and the Short Form (SF-36). A total of 62 patients were included (n = 51 female, mean age 61 (SD 13) years). According to HADS, the prevalence of anxiety was 45% in the postoperative and 42% in the wait-and-watch group (*p* = 0.60), and depression was 61% and 87%, respectively (*p* = 0.005). In total, 43% of patients under wait-and-watch and 37% of patients in the postoperative group scored ≥6 on the DT scale. SF-36 scores were similar in all categories except general health (*p* = 0.005) and physical component aggregate score (43.7 (13.6) vs. 50.5 (9.5), (*p* = 0.03), both lower in the wait-and-watch group. Multivariate analysis revealed the wait-and-watch strategy was associated with a 4.26-fold higher risk of a pathological depression score based on HADS (*p* = 0.03). This study demonstrates a high prevalence of psychological distress in meningioma patients. Further evaluation is necessary to identify the patients in need of psychooncological support.

## 1. Introduction

Meningiomas are among the most commonly diagnosed primary central nervous system tumors, accounting for approximately one-third of all such cases [1]. They are usually benign and slow growing lesions, more often diagnosed in elderly patients and women [2]. Asymptomatic meningiomas can be found in 1–2% of cranial MRIs [3,4]. Due to the increasing availability of diagnostic imaging, the number of patients with meningiomas as an incidental finding has increased. The majority of such neoplasms demonstrate only minimal growth; therefore, a wait-and-watch or conservative strategy can be undertaken until the lesion is significantly larger or becomes symptomatic [5]. The consultation of meningioma patients harboring incidental meningiomas regarding surgery or the wait-and-watch strategy is challenging and requires sensitive skills. 

At the same time, the diagnosis of a brain tumor is generally associated with significant physical and psychosocial burden regardless of tumor entity [6]. Together with cognitive and performance status, psychological well-being is one of the main predictors of the health-related quality of life (HRQoL) [7]. Meningioma patients report worse HRQoL than healthy controls after surgery, and even though it is better than in glioma patients, this difference between brain tumor patient groups is not clinically relevant [8]. However, to our knowledge, most of the studies assessed brain tumor patients who underwent surgery: The HRQoL and psychological distress decrease after the operation, but the data on whether it comes back to normal, are conflicting [9,10].

In contrast, there are few data on the psychological burden and HRQoL of brain tumor patients under a wait-and-watch strategy without histopathologically confirmed diagnoses. A patient with a small asymptomatic meningioma faces a brain tumor diagnosis and—in most of the cases—a recommendation for a follow-up MRI without any further supportive measures such as psychooncological support. These patients are seen less frequently, usually once every year or two, as recommended in the guidelines [11]. There is no meningioma-specific HRQoL questionnaire and, in contrast to glioma patients, there is no established distress screening. The identification of distressed patients in this group could help evolve better strategies for patient follow-up, treatment, and support.

The aim of the study was to investigate the psychological burden in patients with meningiomas with a recommendation of wait-and-watch compared to patients with a good outcome after complete resection.

## 2. Results

### 2.1. Patients

A total of sixty-two patients (51 (82%) female, mean age 61 years (standard deviation (SD) 13)) participated in the study. The detailed patient characteristics are provided in Table 1. The wait-and-watch and postoperative groups were relatively comparable, with about 36% of patients having convexity meningiomas. There was a similar female-to-male ratio and proportion of patients living with a partner. The patients in the wait-and-watch group were slightly older (66 (SD 12) vs. 57 (SD 13) years, *p* = 0.008). More than 90% of patients assessed had an Eastern Cooperative Oncology Group Performance Status (ECOG) 0 or 1 in both groups. The mean Neurologic Assessment in Neuro-Oncology (NANO) score was 0.4 (SD 0.9, range 0-4) and was similar in both study groups. There were 14 (23%) patients in total who were given 1 or more points. There were two (6%) patients under wait-and-watch and one (3%) patient in the postoperative group diagnosed with a psychiatric disorder. There was a significant difference in meningioma size before the operation in comparison with patients in the wait-and-watch group (30 mm (SD 18) and 18 mm (SD 10), respectively, *p* = 0.016). The mean time since the meningioma diagnosis in the wait-and-watch group vs. the time since the operation was comparable (39 (SD 47) and 32 (SD 44) months, respectively). Tumor growth was seen in six (19%) patients in the wait-and-watch group. A tumor relapse was diagnosed in two (6%) patients after meningioma operation. There were four (15%) WHO II° meningiomas; however, no patient received radiotherapy or alternative treatment methods in either group. The use of steroid medications was not covered during the interview.

### 2.2. Psychological Burden as Measured by the HADS and DT

**Figure 1:**
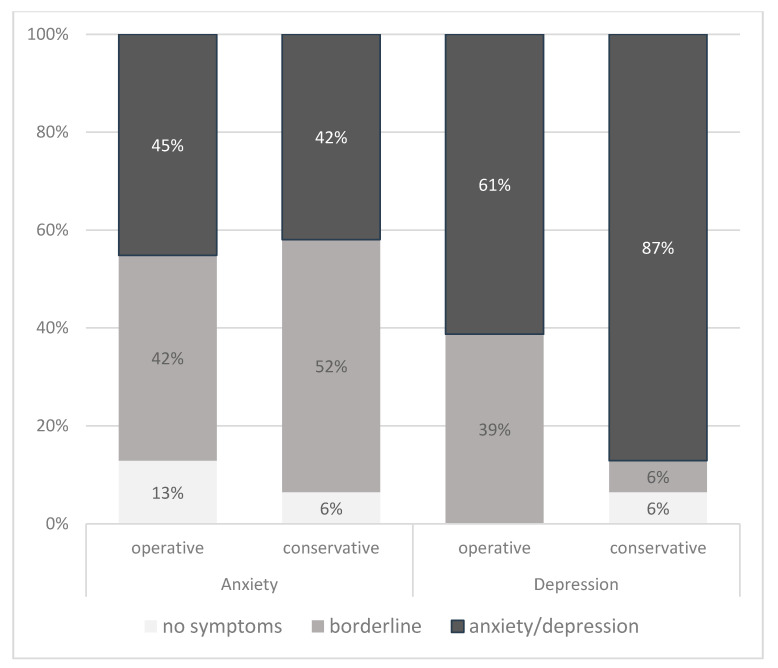
Distribution of patients demonstrating borderline and pathological values on the HADS scores among study groups. A score less than 8 is considered normal, 8–10 as borderline, and higher than 10 as pathological.

The mean Hospital Anxiety and Depression Scale anxiety (HADS-A) score was 10.0 (SD 2.0, range 4–14) in the wait-and-watch and 10.0 (SD 1.8, range 6–14, *p* = 0.92 in the postoperative group. The HADS depression (HADS-D) score was 11.3 (SD 1.9, range 4–14) and 10.9, respectively (SD 1.5, range 8–13), *p* = 0.23. A majority of patients in both groups scored borderline or pathological values (Figure 1). Such values were identified in 94% of patients in the wait-and-watch group and 87% in the postoperative group on the HADS-A score as well as in 94% and 100% of patients based on the HADS-D score, respectively. No statistically significant difference between the groups was found for the HADS-A scale (*p* = 0.60); however, significantly more patients in the wait-and-watch group demonstrated pathological values on the HADS-D score (*p* = 0.005).

The Distress Thermometer (DT) showed significant psychological distress in a large proportion of both study groups. The mean DT score in the wait-and-watch and operative groups was comparable (4.9 (SD 2.0) vs. 4.1 (SD 2.9), *p* = 0.31). However, 43% of patients under wait-and-watch and 37% of patients in the postoperative group demonstrated significant distress, i.e., a DT value ≥ 6. Analysis of the DT demonstrated a significantly higher proportion of patients in the wait-and-watch group having problems with appearance (19% vs. 0%, *p* = 0.01), mouth sores (29.0% vs. 6.5%, *p* = 0.02), and pain (67.7% vs. 41.9%, *p* = 0.04) (Figure S1). Fatigue was the most common problem identified in the problem list in both study groups (55% in the postoperative group and 48% in the wait-and-watch group), followed by worry (45% and 52%) and sleep disturbances (45% and 42%, respectively).

### 2.3. HRQoL as Measured by the SF-36

**Figure 2:**
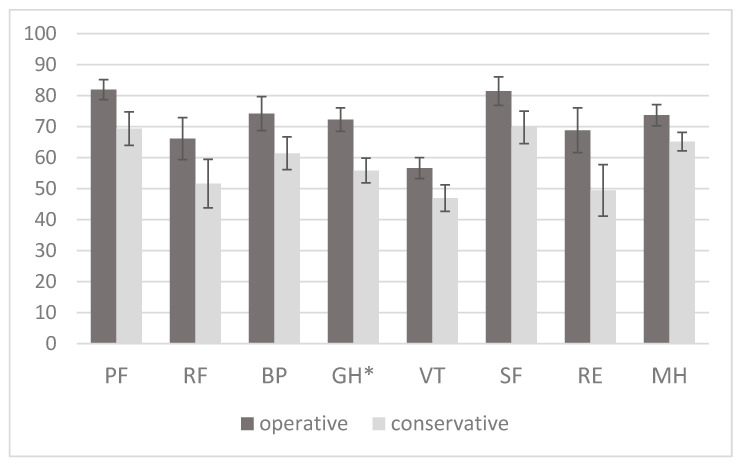
Comparison of the components of the SF-36 Health Survey among study groups. The asterisk represents a significant difference between the groups (*p* < 0.05); error bars represent the standard error of the mean. PF—physical functioning, RP—role-physical, BP—bodily pain, GH—general health, VT—vitality, SF—social functioning, RE—role-emotional, MH—mental health.

The analysis of the Short Form Health Survey (SF-36) results revealed that there was no statistically significant differences in health perception between study groups in all categories except general health, which was significantly lower in the wait-and-watch group (*p* = 0.005) (Figure 2). The physical health component aggregate score was significantly lower in the wait-and-watch group (43.7 (13.6) vs. 50.5 (9.5), *p* = 0.03); the mental health component aggregate score was similar in both groups (39.9 (15.7) vs. 46.2 (13.3), *p* = 0.10). 

### 2.4. Fatigue as Measured by the BFI

The mean total Brief Fatigue Inventory (BFI) score was similar in the wait-and-watch and postoperative groups (3.3 (1.5) vs. 3.4 (1.8), *p* = 0.95). The mean values as well as the proportion of patients with clinically significant fatigue (≥7) were similar among the subscales, except for enjoyment of life, where the wait-and-watch group scored significantly worse values in comparison to the postoperative group (Table 2). The “worst fatigue” was high: 45.2% in the wait-and-watch and 58.1% in the postoperative group. No correlation was found between mean and “worst fatigue” and DT and HADS scores. 

### 2.5. Factors Associated with Higher Psychological Burden

We evaluated the correlation between the results of different measures of psychological distress: HADS-A and HADS-D scores, DT score, Role-Emotional (RE), and aggregate Mental component of SF-36. Among all pairs, significant correlations between the DT score and the Mental component (Spearman’s rho −0.68, *p* < 0.001) and between the DT score and RE (Spearman’s rho −0.57, *p* < 0.001) were found. To identify the factors associated with significant distress, a univariate logistic regression was performed. As there were few patients without symptoms according to the HADS scores, the classification was simplified into two groups: pathologic and borderline/no symptoms. DT ≥ 6 was further used to indicate significant distress. The WHO Grade was not included, as there were too few cases with higher grades. There were no statistically significant associations for pathological DT and HADS-A scores. The wait-and-watch strategy was associated with a 4.26-fold higher risk of a pathological score on the HADS-D score (*p* = 0.03), and the higher (worse) NANO score was associated with a lower risk of depression symptoms (*p* = 0.04) (Table 3). The mean NANO score in patients with depressive symptoms was 0.2 (SD 0.5) and in other patients 1.1 (SD 1.4). Both factors (NANO scores and treatment group) remained significantly associated with depression in HADS-D in a multivariate model (Table 4). 

As the majority of patients (50 out of 62) were included in 2019, the Covid-19 pandemic did not influence the mental health of most patients. A direct comparison among the recruited patients in 2020 and earlier did not show any significant differences.

## 3. Discussion

The data on psychological distress in meningioma patients are considerably low in comparison to more aggressive CNS tumors, such as gliomas. Furthermore, less is known about conservatively treated patients, as the existing studies mostly focus on pre- and postoperative patients. Although this study included only apparently asymptomatic patients with excellent outcome after meningioma operation or under a wait-and-watch strategy, we observed high proportions of significant psychological distress. More than 80% of patients in both groups scored borderline or pathological anxiety values. The values for depression were high in more than 90% of patients in both groups. The wait-and-watch strategy was associated with a higher risk of depression symptoms, and more than one-third of patients in both groups experienced significant distress according to the DT data. 

### 3.1. Depression and Anxiety in Meningioma Patients

The few existing studies on depression and anxiety in meningioma patients demonstrate increased levels of distress in comparison to the general population—the proportion of patients with abnormal HADS-A scores reached 70%, and for HADS-D, 30% in operated patients [12,13,14,15]. In most cases, symptoms declined after the operation. A study examining patients with meningiomas and schwannomas of cerebellopontine angle under a watch-and-wait strategy reported clinically relevant levels of anxiety and depression in one-third of patients [16]. The proportion of anxiety and depression in our cohort was higher in comparison to comparison to other studies [12,13,14,15,16]. This might reflect some regional differences. However, a larger multicenter study is needed to further investigate these observations. The mean values of the HADS score in our study were approximately twice as high as the value of the normal German population [17]. These results are particularly alarming, as a statistically significant difference in the overall survival of meningioma patients stratified according to their preoperative HADS-D score was reported [12]. 

### 3.2. Patients’ Quality of Life

In our study, the values of the physical health component of SF-36 were comparable to the values reported in meningioma patients before and after surgical treatment [13]. After meningioma surgery, patients generally report lower HRQoL compared to healthy controls [8,18], although some data on patients who underwent surgery for asymptomatic meningiomas demonstrated no significant difference compared to the general population [19,20]. On the other hand, data on patients under wait-and-watch are lacking. In a study investigating healthy controls, wait-and-watch patients, and postoperative patients, a lower level HRQoL (by using SF-36) but intact neurocognitive abilities were reported in patients under wait-and-watch [21]. In concordance with our data, wait-and-watch patients reported worse HRQoL compared to the surgical group [21]. A direct comparison between operated and non-operated meningioma patients is difficult, as both treatment strategies cannot be equally offered to each patient. However, the results indicate that the ‘well off’ patients with asymptomatic findings might be suffering from significant psychological burden. Even though only a subscale of General Health and Physical Health Component demonstrated statistically significant differences between study groups, the mean scores of all subscales were lower in the watch-and-wait group. This observation contradicts the ECOG and NANO scales that were similar between the groups, indicating a difference between HRQoL and physical and neurological functioning of the patient, as measured by the examiner. Psychological distress is a strong determinant of HRQoL [7]. There was a significant correlation between SF-36 Role-emotional (RE), mental health component scores, and DT but not with the HADS scores in our study. As each of these scores is a validated instrument, the significance of these differences is difficult to extrapolate and, in our view, stresses many facets of the psychological state of meningioma patients. 

### 3.3. Fatigue

Up to 50% of meningioma patients under wait-and-watch or before the operation suffer from significant fatigue, making it one of the most common symptoms in this patient group [16,22]. Approximately 50% of patients reported being diagnosed with meningioma six months after the onset of symptoms, the most common being headache, fatigue, and cognitive deficit [23]. Meningioma patients reported significantly higher fatigue levels pre- and postoperatively compared to normative values [24]. This is in accordance with our findings, as approximately 50% of patients in both study groups reported significant fatigue. The most common issues identified by patients in our cohort were pain, fatigue, worries, and sleep disturbances. In oncological patients, the presence of fatigue may be disproportionate to the exercised activity and can significantly lower the quality of life [25]. There is no clear explanation why the prevalence of fatigue was high in the wait-and-watch group. Apparently asymptomatic individuals might undergo a diagnostic imaging for variety of reasons, e.g., by participating in research, physician referral, as part of occupational screening or company [26]. These might include a portion of individuals suffering from unspecific symptoms, which include fatigue, anxiety, and depression, who receive cranial imaging. For example, emotional stability is associated with lower depression and anxiety symptom severity in brain tumor patients [27]. On the other hand, the high prevalence of fatigue in postoperative patients with otherwise excellent outcome may become more noticeable once other symptoms subside or reflect the prevalence of per se depressive or anxious individuals getting brain imaging. 

### 3.4. Watch-and-Wait vs. Operative Strategy

The patients in the watch-and-wait group were significantly older than those in the postoperative group (66 vs. 57 years). There was no statistically significant difference in NANO score and ECOG performance status, and age was not a relevant factor for psychological distress. Similar findings were reported previously in glioma patients, where emotional functioning and DT scores were comparable between younger and elderly patients [28].

The psychosocial burden of otherwise successful meningioma surgery can be long lasting. The patients may suffer cognitive, emotional, and social function, fatigue, and sleep impairment for more than 10 years after the operation [22]. Moreover, potentially serious incidental findings require potentially distressful-provoking follow-up [26], which can also apply to postoperative patients getting follow-up imaging. The same assumption might explain the higher HADS and DT scores in this study. Although the fatigue in meningioma patients has been previously associated with anxiety and depression [24], we did not find such a correlation in our patient sample. 

There was no correlation between HADS and DT scores and time since surgery or tumor diagnosis in our study, indicating that the patients may not ‘come to terms’ with a disease even after a significant amount of time. Moreover, the wait-and-watch strategy was associated with depressive symptoms in univariate and multivariate analysis as well as with a lower perception of general health, in SF-36. This was not reflected in observer-based scores (ECOG and NANO)—on the contrary, a worse NANO score was associated with a lower risk of depressive symptoms. This finding might have little clinical value as the difference was less than 1.0 on a scale ranging from 0 to 23 in patients that did not demonstrate meningioma-associated focal neurological deficits. On the other hand, it is well known that clinician-reported outcomes do not always overlap with patient-reported outcomes. Clinicians often report symptoms; however, the symptom might be not relevant for the patients’ HRQoL or psychological well-being [9].

In patients after meningioma operation, the socioeconomic burden might be explained by the employment situation and ability to work [29]. However, in our study, there was no association with employment or relationship status in both study groups. The data on psychological distress patients with WHO II meningiomas cannot be interpreted due to a small number of cases. There was no significant difference in patients with confirmed tumor progress, although these data have to be interpreted cautiously as the number of cases was small. Therefore, it remains unclear how those patients who suffer from relevant distress can be identified. According to a multinational survey of meningioma patients, approximately one-third of the respondents felt that the information received from the health care provider at the time of diagnosis was inadequate and the same proportion of patients received the majority of the information from the internet [23]. This clearly demonstrates that the means of providing information to the patients must be improved. A meningioma-specific questionnaire is urgently needed to address this issue. Nevertheless, routine screenings and psychooncological support might be helpful to reduce distress in patients with even relatively harmless intracranial findings.

### 3.5. Limitations of the Study

There are several limitations to this study. As it was performed in a single tertiary neurosurgical center, a significant selection bias must be taken into account. The patients’ cohorts are not large, which impairs generalizability of the data. Neurocognitive testing was not performed; therefore, the association with HRQoL could not be evaluated. The patients were not asked about current steroid treatment, which might influence mood fluctuations. Furthermore, patients with postoperative morbidity were excluded from the study due to methodological reasons. This may represent a selection bias. Moreover, there was no meningioma-specific questionnaire addressing issues relevant to these patients. Therefore, some of the relevant problems may remain unidentified. 

## 4. Materials and Methods

### 4.1. Study Design and Patients

We performed a monocenter cross-sectional study in the department of neurosurgery in a university hospital. The patients were recruited during their outpatient visits. Two patient groups participated in the study: those under a wait-and-watch strategy and patients with a good postoperative outcome after meningioma resection. The inclusion criteria were (1) age ≥ 18 years, (2) ability to provide informed consent, (3) agreement to participate, and (4a) radiological diagnosis of intracranial meningioma for wait-and-watch strategy-patients or (4b) histologically confirmed completely resected meningioma for operated patients. In order to enable comparability of the patients with asymptomatic meningiomas and operated patients in terms of HRQoL, only patients with an excellent postoperative outcome, i.e., without postoperative meningioma- associated neurological deficit or symptoms (except mild headache (1–2/10 on a numerical analog scale for pain), postoperative scalp hypesthesia or forehead muscle weakness) were included. Exclusion criteria were diagnosis of another tumor, indication for the operation due to a large tumor mass, midline shift, hydrocephalus or neurologic deficits. 

The ethics committee of Rhineland-Palatinate, Germany reviewed and approved this study (2018-13828). All procedures performed were in accordance with the ethical standards of the institutional and national research committee and with the 1964 Helsinki declaration and its later amendments or comparable ethical standards.

### 4.2. Investigations

After signing a consent form, a patient assessment of neurological function using the Neurologic Assessment in Neuro-Oncology (NANO) scale [30] and general performance status Eastern Cooperative Oncology Group (ECOG) Performance Status was performed, and demographic and tumor-specific data were acquired. The demographic data included gender, age, level of education (higher than secondary vs. other), employment, family status (living with a partner), and comorbidities. The patients were explicitly asked about psychiatric comorbidities and active or finished treatment. Tumor localization, size, growth, and histological grade (for the operated patients) were taken into account. The tumor localization was classified into convexity, falx, anterior, middle, posterior fossa, and sella/sinus cavernosus. The time since the last significant event, i.e., tumor diagnosis for the patients under wait-and-watch and time since operation for the operated patients, was also recorded.

### 4.3. Applied Questionnaires

German adaptations of SF-36, DT, HADS, and BFI were used for the evaluation of HRQoL and psychological distress. 

The SF-36 [31,32] is a validated multidimensional questionnaire measuring HRQoL it consists of eight scales describing vitality, physical functioning, bodily pain, general health perceptions, physical role functioning, emotional role functioning, social role functioning, and mental health. Physical and component aggregate scores can be calculated using the aforementioned scales.

The HADS is a questionnaire measuring depressive and anxiety symptoms, based on 14 questions [33,34]. The questions interchangeably assess anxiety and depression and provide two scores: Anxiety score (HADS-A) and Depression score (HADS-D). A score less than 8 is considered normal, 8–10 as borderline, >10 as pathological. 

The DT is an ultrashort screening questionnaire assessing the psychological burden (=“distress”) on a numerical analog scale, 0–10. It is accompanied by a 40-item encompassing problem list with emotional, practical, physical, and spiritual concerns and is validated for brain tumor patients [35]. According to Goebel et al [35], a score of ≥6 on the DT scale is considered a significant burden. 

BFI [36,37] is a questionnaire assessing fatigue based on 10 questions and a mean score. The ‘worst fatigue’ of ≥7 corresponds to clinically significant fatigue. Fatigue severity on the BFI scale of 0–6 is considered “non-severe” and ≥7, “severe”.

### 4.4. Statistics

The primary outcome was the difference between groups regarding the psychological burden as measured by the HADS score. The sample size was estimated as 31 patients/group, considering no difference in the HADS values between the groups as a null hypothesis, for a clinically relevant difference of ±3; if the standard deviation is not higher than 4, the maximum possibility of type I error = 5% and that of type II error = 20%. Categorical data were described by absolute and relative frequencies, and continuous data were described by the mean and standard deviation. 

Secondary outcomes were the level of psychological distress as measured by the DT, SF-36, and BFI scores and their association with demographic and tumor-associated factors. The difference in the absolute values of the scores between the groups was assessed, after assessing the distribution of the tested variables by Student’s t-test or the Mann–Whitney test. The difference in the distribution in categorical variables was assessed by a Chi-squared test. The correlation between the scores measuring psychological distress (DT, HADS-A, HADS-D, and the mental component of SF-36) was intended to evaluate interchangeability and was assessed using Spearman’s rho. Univariate and multivariate logistic regressions were used to evaluate the association of clinical characteristics with significant psychological burden. For regression analysis, tumor localization was further classified as falx/convexity vs. scull base, and patient age was classified as ≥65 years vs. younger. Gender, family, and employment status, the ECOG performance score and NANO score, tumor size, location, time since last significant event (tumor diagnosis or operation), WHO Grade, and tumor progress were also included in the univariate analysis. No correction for multiple testing was performed. A *p*-value less than 0.05 was considered statistically significant.

## 5. Conclusions

This study demonstrates a high prevalence of psychological distress in meningioma patients independent of management strategy. Further evaluation is necessary to identify the patients in need of psychooncological support. 

## Figures and Tables

**Table 1:** Patient and tumor characteristics.

		Postoperative	Wait-and-Watch	Total	
**N**		31	31	62	
**Age (SD)**		57 (13)	66 (12)	61 (13)	*p* = 0.008
**Female, %**		25 (81%)	26 (84%)	51 (82%)	
**Family situation, %**					
	living with a partner	22 (71%)	18 (62%)	40 (67%)	
	living alone	9 (29%)	11 (38%)	20 (33%)	
**Employment, %**					*p* < 0.001
	Full	12 (39)	9 (31%)	21 (35%)	
	part-time	2 (6%)	0 (0%)	2 (3%)	
	Unemployed	6 (19%)	0 (0%)	6 (10%)	
	Retired	11 (35%)	20 (69%)	31 (52%)	
**ECOG, %**					
	0	24 (77%)	26 (84%)	50 (81%)	
	1	7 (23%)	2 (6%)	9 (15%)	
	2	0 (0%)	3 (10%)	3 (5%)	
**NANO scale, mean (SD)**		0.5 (1.0)	0.4 (0.8)	0.4 (0.9)	
**Psychiatric disorder**		1 (3%)	2 (6%)	3 (5%)	
**Tumor localisation, %**					
	Convexity	10 (36%)	11 (37%)	21 (36%)	
	Falx	4 (14%)	5 (17%)	9 (15%)	
	Anterior fossa	4 (14%)	2 (7%)	6 (10%)	
	Middle fossa	2 (7%)	5 (7%)	7(12%)	
	Posterior fossa	5 (18%)	4 (13%)	9 (15%)	
	Sella/sinus cavernosus	3 (11%)	2 (7%)	5 (9%)	
**WHO histological grade**					
	Grade I	27 (87%)			
	Grade II	4 (13%)			
**Time after diagnosis, months (SD)**			39 (47)		
**Time after operation, months (SD)**		32 (44)			
**Tumor size, mm**		30 (18)	18 (10)		*p* = 0.004
**Tumor growth**		2 (6%)	6 (19%)		

NANO—Neurologic Assessment in Neuro-Oncology scale, ECOG—Eastern Cooperative Oncology Group Performance Status. *p*-values for statistically significant differences (*p* < 0.05) between the groups are provided.

**Table 2:** The mean scores of the Brief Fatigue Inventory and the proportion of patients scoring ≥7 in study groups. The asterisk indicates a statistically significant difference between the groups.

	Mean (SD)		Proportion with Significant Fatigue (≥7)	
Subscales	Postoperative	Wait-and-Watch	Postoperative	Wait-and-Watch
Fatigue right now	3.5 (2.2)	3.8 (2.3)	12.9%	9.7%
Usual fatigue	3.7 (1.9)	4.2 (2.3)	6.5%	19.4%
Worst fatigue	6.2 (2.9)	5.6 (2.5)	58.1%	45.2%
Activity	3.4 (3.4)	3.4 (2.5)	12.9%	12.9%
Mood	2.8 (1.9)	3.4 (2.3)	6.5%	12.9%
Walking	2.2 (1.8)	2.8 (1.9)	6.5%	12.9%
Working	2.6 (2.0)	3.4 (2.3)	3.2%	6.5%
Relation to others	2.2 (2.0)	2.7 (2.3)	6.5%	12.9%
Enjoyment of life	2.2 (2.0) *	3.5 (2.3) *	6.5%	16.1%
**Mean score**	3.4 (1.8)	3.3 (1.5)	6.5%	3.4%

**Table 3:** Association of patient- and tumor-associated factors with significant psychological distress. OR—odds ratio, 95% CI—confidence interval, DT ≥ 6—Distress thermometer score of 6 and higher, HADS-A ≥ 11—Anxiety score of 11 and higher in HADS, HADS-D ≥ 11—Depression score of 11 and higher in HADS. Significant fatigue—worse fatigue ≥ 7. The asterisk marks statistically significant values.

	OR (95%CI)		
Variable	DT ≥ 6	HADS-A ≥ 11	HADS-D ≥ 11
Gender	1.24 (0.30–5.18)	1.44 (0.37–5.53)	0.91 (0.21–3.96)
Age ≥ 65 years	1.49 (0.52–4.28)	1.23 (0.44–3.38)	0.50 (0.15–1.66)
Family status	0.41 (0.12–1.36)	0.82 (0.27–2.42)	0.54 (0.17–1.76)
Employment	0.81 (0.55–1.19)	0.96 (0.66–1.39)	0.48 (0.14–1.71)
Education	-	0.36 (0.03–4.25)	1.40 (0.12–16.58)
ECOG 1 vs.0	1.22 (0.29–5.13)	1.02 (0.24–4.25)	0.35 (0.81–1.54)
NANO Score	0.99 (0.23–1.97)	1.13 (0.64–1.98)	0.34 (0.16–0.70) *
Significant fatigue	2.50 (0.86–7.31)	0.60 (0.22–1.65)	1.53 (0.49–4.81)
Wait-and-watch vs. operative treatment	1.32 (0.47–3.72)	0.88 (0.32–2.40)	4.26 (1.19–15.25) *
Tumor location	0.68 (0.23–1.97)	0.62 (0.22–1.80)	0.96 (0.30–3.14)
Tumor size	0.99 (0.96–1.03)	0.98 (0.97–1.03)	0.99 (0.96–1.03)
Time since diagnosis/operation	1.00 (0.99–1.01)	0.99 (0.99–1.01)	0.99 (0.98–1.01)
Tumor progress	1.14 (0.23–5.63)	0.75 (0.16–3.46)	2.69 (0.31–23.78)

**Table 4:** Multivariate model for the clinically significant depression scores. OR—odds ratio, 95% CI—confidence interval, HADS-D ≥ 11—Depression score of 11 and higher in HADS. The asterisk marks statistically significant values. Treatment strategy: wait-and-watch vs. postoperative.

	OR (95%CI)
Variable	HADS-D ≥ 11
NANO Score	0.27 (0.11–0.65) *
Wait-and-watch vs. operative treatment	5.83 (1.12–28.85) *

## Supplementary Materials

The following are available online at https://www.mdpi.com/2072-6694/12/12/3503/s1, Figure S1: Proportion of positive answers to the Distress Thermometer Problem List in postoperative and wait-and-watch groups. Asterisk marks significant difference between the groups (*p* < 0.05).
