# Supplementary material for: Psychological Burden in Meningioma Patients under a Wait-and-Watch Strategy and after Complete Resection Is High—Results of a Prospective Single Center Study
Source: Cancers (Basel). 2020 Nov 25;12(12):3503. doi: 10.3390/cancers12123503 (PMC7761113; doi:10.3390/cancers12123503)
Supplement: Supplementary file 1 [file cancers-12-03503-s001.pdf]

Supplementary Materials:

Psychological Burden in Meningioma Patients Under a Wait-and-Watch Strategy and After Complete Resection Is High—Results of a Prospective Single Center Study

Darius Kalasauskas [1,*], Naureen Keric [1], Salman Abu Ajaj [1], Leoni von Cube [1], Florian Ringel [1] and Mirjam Renovanz [1,2,3]

[1] Department of Neurosurgery, University Medical Centre, Johannes Gutenberg University Mainz, Langenbeckstr. 1, 55131 Mainz, Germany; Naureen.Keric@unimedizin-mainz.de (N.K.); sabuajaj@students.uni-mainz.de (S.A.A.); lvoncube@students.uni-mainz.de (L.v.C.); Florian.Ringel@unimedizin-mainz.de (F.R.); mirjam.renovanz@med.uni-tuebingen.de (M.R.)

[2] Department of Neurosurgery, University Hospital Tübingen, Eberhard Karls University Tübingen, Hoppe-Seyler-Straße 3, 72076 Tübingen, Germany

[3] Department of Neurology & Interdisciplinary Neuro-Oncology, University Hospital Tübingen, Hertie Institute for Clinical Brain Research, Otfried-Müller-Straße 27, 72076 Tübingen, Germany

[*] Correspondence: Darius.Kalasauskas@unimedizin-mainz.de; Tel.: +49-6131-177331; Fax: +49-6131-172274

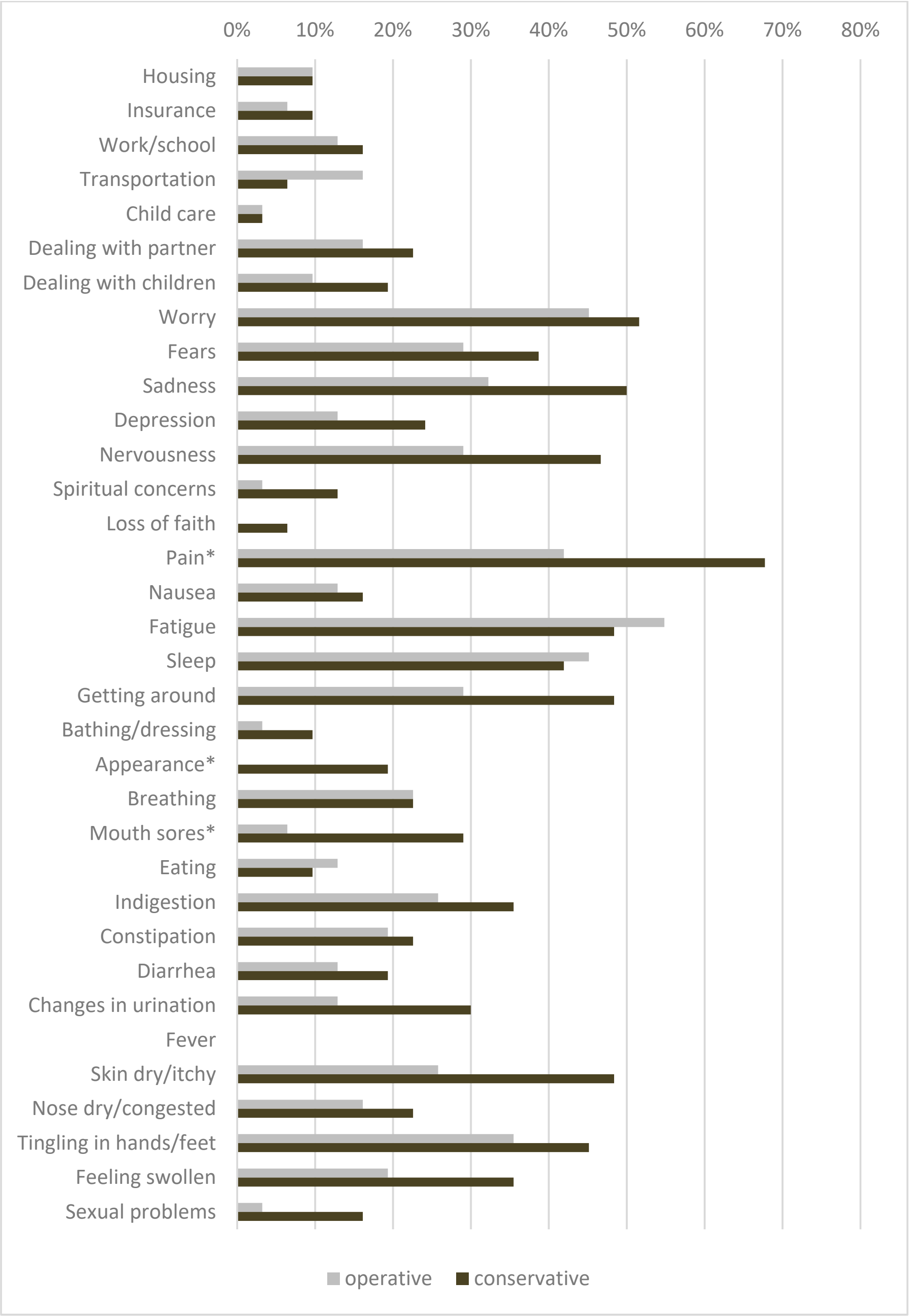

Figure S1. Proportion of positive answers to the Distress Thermometer Problem List in postoperative and wait-and-watch groups. Asterisk marks significant difference between the groups ($p < 0.05$).
